# Outcomes and comorbidities of Merkel cell carcinoma: A retrospective cohort study utilizing the Mayo Clinic platform

**DOI:** 10.1016/j.jdin.2026.06.011

**Published:** 2026-06-26

**Authors:** Sarah A. Amjad, Jenna E. Koblinski, Micah Starghill, Samantha Shao, Jacob Breinholt, James W. Jakub, Christopher J. Arpey, Christian L. Baum, Phillip Pirgousis, Roxana S. Dronca, Aaron R. Mangold, Collin M. Costello

**Affiliations:** a Mayo Clinic Alix School of Medicine, Jacksonville, Florida; b Department of Dermatology, Emory, Atlanta, Georgia; c Mayo Clinic Alix School of Medicine, Phoenix, Arizona; d Division of Surgical Oncology, Mayo Clinic, Jacksonville, Florida; e Department of Dermatology, Mayo Clinic, Scottsdale, Arizona; f Department of Dermatology, Mayo Clinic, Rochester, Minnesota; g Department of Otolaryngology, Mayo Clinic, Jacksonville, Florida; h Division of Hematology and Medical Oncology, Mayo Clinic, Jacksonville, Florida

**Keywords:** comorbidities, health care utilization, hospitalizations, immunosuppression, malignancy, Merkel cell carcinoma, mortality, neuroendocrine tumor, outcomes, polyomavirus, skin cancer, survival, visceral cancer

*To the Editor:* Merkel cell carcinoma (MCC) is an aggressive neuroendocrine tumor with significant mortality risk, and the incidence of MCC has risen exponentially over the last 30 years.^1^ Prior studies using national cancer databases have associated MCC with hematologic and solid organ malignancies but have been limited by available data.^2^^,^^3^ Our aim was to compare outcomes and comorbidities among patients with MCC, those without MCC, and those with other malignancies.

We performed a retrospective, single-enterprise–cohort study. The inclusion criteria for the MCC cohort included a diagnosis of MCC at least once. The non-MCC cohort and malignancy cohorts comprised propensity-matched patients without a diagnosis of MCC and a diagnosis of the corresponding malignancy, respectively. Our outcome end points were the number of hospitalizations, critical care unit (CCU) admissions, overall mortality, and short- and long-term survival. See Supplementary Methods I (available via Mendeley at https://data.mendeley.com/datasets/xdt6hdvyfx/1) for further details.

The study included 1092 patients diagnosed with MCC. In the largest MCC cohort, the majority of patients were White (91.6%) and non-Hispanic (83.8%) males (66.4%) with a mean age of 72 years. Further patient characteristics prepropensity and postpropensity-matching for MCC versus non-MCC cohorts and melanoma cohorts are summarized in Table I and Supplementary Table I (available via Mendeley at https://data.mendeley.com/datasets/xdt6hdvyfx/1), respectively.

**Table I:** Prepropensity and postpropensity matched cohorts with Merkel cell carcinoma versus non–Merkel cell carcinoma demographics

Prepropensity-matched characteristics				Postpropensity-matched characteristics			
	MCC, *n* = 1092 (%)	Non-MCC, *n* = 7,519,250 (%)	*P*∗		MCC, *n* = 1044 (%)	Non-MCC, *n* = 1044 (%)	*P*∗
Age, y				Age, y			
Mean age	71.9	43.3		Mean age	72	72.1	
Sex				Sex			
Male	723 (66.2)	3,589,867 (47.7)	<.001	Male	693 (66.4)	693 (66.4)	1
Female	369 (33.8)	3,927,214 (52.2)	<.001	Female	351 (33.6)	351 (33.6)	1
Unknown	0 (0)	1890 (0.03)	.6	Unknown	0 (0)	0 (0)	1
Race				Race			
White	999 (91.5)	4,457,677 (59.3)	<.001	White	956 (91.6)	949 (90.9)	.59
Black	4 (0.4)	217,490 (2.9)	<.001	Black	4 (0.4)	9 (0.9)	.16
Asian	6 (0.6)	115,254 (1.5)	.048	Asian	5 (0.5)	7 (0.7)	.45
NAPI	6 (0.5)	37,226 (0.5)	.8	NAPI	5 (0.5)	9 (0.9)	.28
Unknown	77 (7.1)	2,691,324 (35.8)	<.001	Unknown	74 (7.1)	69 (6.6)	.66
Ethnicity				Ethnicity			
Hispanic	23 (2.1)	239,704 (3.2)	.042	Hispanic	22 (2.1)	30 (2.9)	.26
Non-Hispanic	916 (83.9)	3,678,152 (48.9)	<.001	Non-Hispanic	875 (83.8)	870 (83.3)	.77
Unknown	153 (14)	3,601,115 (47.9)	<.001	Unknown	147 (14.1)	144 (13.8)	.85

*MCC*, Merkel cell carcinoma; *NAPI*, Native American/Pacific Islander.

∗ Pearson χ^2^ test *P* value.

We found a significant increase in hospitalizations and CCU admissions in the MCC cohort compared with the non-MCC cohort, with rates of 53.5% versus 31.6% and 13.1% versus 7.3%, respectively (*P* < .0001). There was a significant increase in overall mortality and a decrease in short- and long-term survival in the MCC cohort compared with the non-MCC cohort (*P* < .0001). See Supplementary Results I (available via Mendeley at https://data.mendeley.com/datasets/xdt6hdvyfx/1) for further details. Characterization of all outcomes, including survival differences between MCC and the other cutaneous and solid organ tumors studied, is described in Table II and Supplementary Figure I (available via Mendeley at https://data.mendeley.com/datasets/xdt6hdvyfx/1).

**Table II:** Outcomes in patient cohorts with Merkel cell carcinoma versus other cutaneous and solid organ malignancies

Column 1	MCC, *n* = 1027 (%)	Melanoma, *n* = 1027 (%)	*P*∗ value	MCC, *n* = 1039 (%)	Lung cancer, *n* = 1039 (%)	*P*∗ value	MCC, *n* = 1036 (%)	Pancreatic cancer, *n* = 1036 (%)	*P*∗ value	MCC, *n* = 1022 (%)	CRC, *n* = 1022 (%)	*P*∗ value	MCC, *n* = 1029 (%)	Breast/Prostate, *n* = 1029 (%)	*P*∗ value	MCC, *n* = 938 (%)	Glioblastoma, *n* = 938 (%)	*P*∗ value	MCC, *n* = 999 (%)	CTCL, *n* = 999 (%)	*P*∗ value
Hospitalization no. (%)	548 (53.4)	507 (49.4)	.07	553 (53.2)	665 (64)	<.001	553 (53.4)	570 (55.0)	.45	533 (52.2)	623 (61.0)	<.001	541 (52.6)	455 (44.2)	<.001	490 (52.2)	599 (63.9)	<.001	531 (53.2)	451 (45.1)	<.001
CCU no. (%)	123 (12.9)	125 (12.2)	.64	134 (12.9)	209 (20.1)	<.001	134 (12.9)	142 (13.7)	.61	124 (12.1)	156 (15.3)	.04	127 (12.3)	99 (9.6)	.05	121 (12.9)	129 (13.8)	.59	125 (12.5)	131 (13.1)	.69
Deceased no. (%)	420 (40.9)	332 (32.3)	<.001	427 (41.1)	650 (62.6)	<.001	427 (41.2)	648 (62.5)	<.001	338 (33.1)	398 (38.9)	.01	338 (32.8)	217 (21.1)	<.001	319 (34)	619 (66)	<.001	333 (33.3)	345 (34.5)	.57
60-Mo survival (%)	59.3	75.7	<.001	59	37.1	<.001	58.8	28.5	<.001	63.4	62.6	.67	63.6	84.5	<.001	63.8	10.3	<.001	63.6	68	.04
120-Mo survival (%)	46.7	58.4	<.001	46.1	22.5	<.001	46.1	22.3	<.001	53.1	50.6	.45	53.3	71.6	<.001	53	7.4	<.001	53.3	52.2	.19
12-Mo survival (%)	82.8	92.2	<.001	82.5	64.3	<.001	82.5	57.8	<.001	83.5	81.8	.30	83.7	95.1	<.001	83.7	44.4	<.001	83.4	86.1	.11
18-Mo survival (%)	78	89.6	<.001	77.9	57.9	<.001	78	48.4	<.001	79.2	78	.43	79.5	93.5	<.001	79.1	32.6	<.001	79.1	82.1	.12
24-Mo survival (%)	73.7	88	<.001	73.4	52.3	<.001	73.5	44.1	<.001	74.7	75	.83	74.9	91.5	<.001	74.5	23.8	<.001	74.4	79.2	.03

*CRC*, Colorectal carcinoma; *CTCL*, cutaneous T-cell lymphoma; *MCC*, Merkel cell carcinoma.

∗ Fisher exact *P* value.

When comparing the MCC versus non-MCC cohort, patients with MCC were more likely to have hematologic malignancies or disorders, immunodeficiencies, skin cancers, and other solid organ tumors (Supplementary Tables II and III, available via Mendeley at https://data.mendeley.com/datasets/xdt6hdvyfx/1). They were also more likely to have various cardiovascular, hepatic, renal, and pulmonary diseases, as further described in Supplementary Table IV (available via Mendeley at https://data.mendeley.com/datasets/xdt6hdvyfx/1).

Our study supports that MCC is associated with decreased survival, increased morbidity, and increased health care utilization due to the higher comorbidity burden. Notably, we examined CCU admissions and survival timelines across cohorts and further contextualized our results by comparing them with other malignancies. Few studies have compared health care utilization between MCC and other cancers, but none have investigated hospitalization rates, CCU admission rates, and survival outcomes in MCC versus other cancers.^4^^,^^5^ Our study suggests that malignancies such as breast/prostate cancer and cutaneous T-cell lymphoma have decreased hospital utilization and improved survival outcomes compared with MCC, while lung cancer, colorectal cancer, and glioblastoma have increased hospital utilization and poorer outcomes compared with MCC. Our data was limited to a single electronic health record; thus, we were unable to obtain propensity-matched patients based on immunosuppression status and disease stage, isolate cancer-related hospitalizations, and provide disease-specific mortality. Data describing health care utilization in MCC are scarce, and more research is needed to compare MCC health care utilization with that of other cancers.

## Conflicts of interest

Dr Koblinski is an investigator for Merck (unrelated to this study) and has no relevant conflicts of interest to declare. Dr Costello is an investigator for Merck (unrelated to this study) and was an investigator for DeepX Health within the last 12 months (unrelated to this study) and has no relevant conflicts of interest to declare. Dr Mangold has consulted for Phlecs BV, Kyowa, Eli Lilly, Momenta, UCB, and Regeneron in the past, more than 24 months ago; he has consulted for Incyte, Soligenix, Clarivate, Argenyx, and Bristol Myers Squibb in the past, less than 12 months ago; he consults for Leo Pharma, PPD, Mallinckrodt, Nuvig, Tourmaline Bio, Janssen, Boehringer Ingelheim, and Biocryst currently; he consults for Regeneron and Pfizer, currently with payments to the institution; and he has grant support from Kyowa, Miragen, Regeneron, Incyte, Eli Lilly, Argenx, Palvella, AbbVie, Priovant, Bristol Myers Squibb, Merck, Sci-Tech, Soligenix, Insmed, and AstraZeneca in the last 24 months. Beyond 24 months, grant support has come from Sun Pharma, Elorac, Novartis, Janssen, Miragen, and Corbus. He has received royalties from Priovant, Adelphi Values, and Clarivate. His current patents include Methods and Materials for Assessing and Treating Cutaneous Squamous Cell Carcinoma (PCT/US2023/078902), Use of Oral Jaki in Lichen Planus (PCT/US2024/020149), Topical Ruxolitinib in Lichen Planus (PCT/US2021/053149, 2023-520085, and 21805700.8, respectively), Methods and Materials for Treating Lichen Planopilaris (Registration Number: 53,103), and Machine-Learning Models for Tumor Grading Using Rank-Aware Contextual Reasoning on Whole Slide Images (63/616,287). Authors Amjad, Starghill, Shao, and Breinholt, and Drs Jakub, Arpey, Baum, Pirgousis, and Dronca have no conflicts of interest to declare.
